# Excipient knowledgebase: Development of a comprehensive tool for understanding the disposition and interaction potential of common excipients

**DOI:** 10.1002/psp4.12668

**Published:** 2021-08-01

**Authors:** Savannah J. McFeely, Jingjing Yu, Yan Wang, Cheryl Wu, Isabelle Ragueneau‐Majlessi

**Affiliations:** ^1^ Department of Pharmaceutics School of Pharmacy UW Drug Interaction Solutions University of Washington Seattle WA USA; ^2^ Present address: Certara UK, Simcyp Division Sheffield UK

## Abstract

Although the use of excipients is widespread, a thorough understanding of the drug interaction potential of these compounds remains a frequent topic of current research. Not only can excipients alter the disposition of coformulated drugs, but it is likely that these effects on co‐administered drugs can reach to clinical significance leading to potential adverse effects or loss of efficacy. These risks can be evaluated through use of in silico methods of mechanistic modeling, including approaches, such as population pharmacokinetic (PK) and physiologically‐based PK modeling, which require a comprehensive understanding of the compounds to ensure accurate predictions. We established a knowledgebase of the available compound (or substance) and interaction‐specific parameters with the goal of providing a single source of physiochemical, in vitro, and clinical PK and interaction data of commonly used excipients. To illustrate the utility of this knowledgebase, a model for cremophor EL was developed and used to hypothesize the potential for CYP3A‐ and P‐gp‐based interactions as a proof of concept.

**Study Highlights**

**WHAT IS THE CURRENT KNOWLEDGE ON THE TOPIC?**

Although widely used, there are few references that provide comprehensive data on excipients and how they might affect drug metabolism and transport.

**WHAT QUESTION DID THIS STUDY ADDRESS?**

This study introduces a novel knowledgebase of modeling‐focused parameters for commonly used excipients.

**WHAT DOES THIS STUDY ADD TO OUR KNOWLEDGE?**

The knowledgebase serves as a comprehensive repository for the current research concerning excipients, highlighting not only the parameters that are well‐defined but also those that would benefit from additional research.

**HOW MIGHT THIS CHANGE DRUG DISCOVERY, DEVELOPMENT, AND/OR THERAPEUTICS?**

Having a single source where chemical and pharmacokinetic parameters can be found for excipients of interest facilitates modeling endeavors to better predict clinically meaningful excipient‐drug interactions.

## INTRODUCTION

Excipients are a unique class of compounds that represent an often overlooked source of potential pharmacokinetic (PK) interactions. Although it is widely accepted that excipients offer solutions to formulation challenges, including low solubility and bioavailability, the understanding of the various mechanisms by which these compounds could alter drug disposition remains limited. It is likely that the mechanisms responsible for the desirable changes in PKs can also contribute to changes in the drug‐drug interaction (DDI) profile of the coformulated active compound but also of co‐administered drugs, due to alterations in enzyme and/or transporter function. Chen et al., for example, reported recently an unexpected and complex DDI between itraconazole and fenebrutinib that was explained by the effect of hydroxypropyl‐β‐cyclodextrin contained in itraconazole oral solution on the absorption of fenebrutinib, partially masking the change in fenebrutinib disposition related to inhibition of its CYP3A‐mediated metabolism by itraconazole.^1^ Excipient‐drug interactions (EDIs) at the level of metabolism and transport has long been a neglected area but just received increased attention in recent years.^2^, ^3^ Thus, characterizing such potential will allow optimization of formulation design and dose selection. For these reasons it is critical to develop a better mechanistic understanding of the possible EDIs based on a systematic and quantitative analysis of the research data available in the literature.

Recent years have seen a dramatic increase in the utilization of in silico methods, such as physiologically‐based pharmacokinetic (PBPK) modeling, to predict the disposition of drugs in various patient populations and predict the risk of interactions during the drug development process. In these computational models, it is paramount to include accurate estimates of drug‐specific parameters in the simulation trials and compare, when practical, to observed clinical data. These parameters, both compound‐specific and clinical observations, are typically acquired from many sources (such as new drug application reviews and peer‐reviewed publications), requiring significant time to identify and compile the required data. Therefore, curating and extracting these parameters, while simultaneously analyzing the reliability and reported variability, to develop a PBPK‐DDI/EDI knowledgebase that can be used at any stage of research is paramount to support quantitative predictions and advance understanding in this area.

To this end, it was determined that inclusion of common excipients in a knowledgebase would (i) allow for the identification of relevant excipient‐specific parameters and PK‐based EDI data currently available in literature that are required for better understanding the potential enzyme‐, transporter‐, or other mechanism‐mediated interactions for these excipients and (ii) identify knowledge gaps, highlighting those parameters where more research is needed to better understand the disposition and interaction potential. Additionally, development of a central repository for this information would allow for efficient and accurate modeling development to facilitate collaborative research into better understanding these excipients.

## METHODS

### Knowledgebase construction and validation

A list of 37 commonly utilized excipients was developed for initial evaluation in the knowledgebase (Table 1). Excipients were chosen based on frequency of use among formulation experts, those serving as solubility enhancing agents, availability in the US Food and Drug Administration’s (FDA’s) excipient database, and overall predicted availability of data due to time in use. Data was compiled in three main categories—compound parameters, in vitro data (human), and clinical data (including both PK and PK‐based interaction data with the compound as substrate and precipitant). A full listing of the data of interest for each category is included in Table S3, with a general overview presented here.

**TABLE 1:** Summary of data for excipients included in knowledgebase

Compound	No data	Cpd param.	Clinical PK	In vitro substrate		In vitro inhibitor/inducer		Clinical data		
				Metabolism	Transport	Metabolism	Transport	Substrate	Inhibitor	Inducer
Acconon MC8‐2							X (H)			
Beta‐cyclodextrin		X								
Brij S20	X									
Brij−35						X (H)	X (H)			
Capmul MCM							X (H)			
Capmul PG−8							X (H)			
Caprylocaproyl polyoxyl−8 glycerides NF	X									
Capsaicin				X		X (H/D)	X (H)			
Cremophor EL		X	X			X (H)	X (H/D)		X	
Cremophor RH40						X (H)	X (H/D)		X	
D ‐α‐Tocopherol polyethylene glycol 1000 succinate						X (H)	X (H)		X	
Diethylene glycol monoethyl ether EP/NF		X	X							
Gamma‐cyclodextrin		X								
Gelucire 44/14							X (H)			
Glyceryl monolinoleate NF		X								
Glyceryl monooleate (Type 40) NF		X								
Glycol monolaurate (Type I, monoesters >45%) NF	X									
Hydroxypropyl beta cyclodextrin		X	X			X (H)	X (H)		X	
Linoleoyl polyoxyl−6 glycerides NF	X									
Monoketocholate	X									
Myrj 52						X (H)	X (H)			
Oleic acid		X				X (H)	X (H)			
Pluronic 188	X					X (H)	X (H)		X	
Pluronic 407						X (H)	X (H)			
Polyethylene glycol 2000						X (H)				
Polyethylene glycol 300							X (H)			
Polysorbate 40		X				X (H)	X (H)			
Polysorbate 80		X	X			X (H/D)	X (H/D)		X	
Propylene glycol		X				X (H/D)			X	
Propylene glycol dicaprylate/dicaprate NF	X									
Propylene glycol monocaprylate (Type I)	X									
Propylene glycol monolaurate (Type I)	X									
Solutol HS15						X (H)	X (H)			
Soybean lecithin						X (H)				
Sulfobutylether‐beta‐cyclodextrin		X	X							
Taurolithocholate	X									
Thiolated chitosan	X									
Total number of compounds	11	12	5	1	0	16 (H = 16; D = 3)	18 (H = 18; D = 3)	0	7	0

Abbreviations: H, inhibition studies; D, induction studies; PK, pharmacokinetics.

Excipient‐specific parameters include identifying information on the excipient compound (CAS number, trade name[s], etc.) in addition to modeling‐relevant physiochemical properties (molecular weight, pKa, solubility, etc.) and biological parameters (blood‐to‐plasma ratio, protein binding, etc.). To assist in the understanding of excipient disposition and interaction potential, in vitro data on the metabolism and transport of the excipients as well as inhibition/induction data (when excipients were evaluated as perpetrators of DDIs) was compiled. For each excipient, parameters, such as K_m_, K_i_, and EC_50_ were included and all identified study values were included to highlight potential variability. The final data category, clinical data, includes both PK studies and interaction data. The excipient was evaluated as both the substrate and precipitant and all relevant parameters, including, but not limited to, the type of interaction (inhibition, induction, or other mechanisms), dosing information for both substrate and precipitant, PK data including changes from baseline (e.g., area under the curve ratio [AUCR], peak plasma concentration [C_max_] ratio), and the drug metabolizing enzymes/transporters/other mechanisms implicated in the interaction were reported. Data collection was completed in a similar manner to previously published studies with queries conducted for published clinical and in vitro data through repositories, such as PubMed and Embase.^4^ Additionally, databases, such as PubChem and ChemIDPlus, were utilized for identifying physiochemical data.

To ensure the knowledgebase was as complete as possible and that data were accurately captured, a standard operating procedure (SOP) regarding data query and entry was created (literature search SOP available in the Supplementary Methods). Guidelines on method of query, including key word search parameters, as well as the frequency to perform literature search were established. Data were entered as presented by study authors, with data estimated from figures noted as such. All data included in the knowledgebase were extracted from the sources and curated by multiple experienced researchers at University of Washington Drug Interaction Solutions and entries were subsequently validated by a second researcher in the team to ensure accurate reporting. As every effort was made to capture all available data, including studies showing an effect and those showing no effect, those compounds listed in the knowledgebase with no information indicate an absence of data currently available in literature.

## RESULTS

### Knowledgebase content and data findings

At the time of publication, there were data available for 26 excipients out of the 37 of interest (26/37, 70%). A total of 420 entries with data for these excipients from 92 publications have been entered (summary presented in Table 1). The complete knowledgebase is available in the Supplementary Materials. The 26 excipients entered had data available in at least one category (compound parameters, in vitro, clinical PK, or interaction), however, many only had one parameter or data from a single study available. Four excipients had only some form of physiochemical data available (4/26, 15%) with no excipient having more than one parameter available in literature. Among the 26 excipients, 15 (58%) had only in vitro data, five (20%) had data in two categories, and four (15%; cremophor EL, hydroxypropyl beta cyclodextrin, polysorbate 80, and propylene glycol) had data available for all three major categories evaluated. None of the excipients’ DDI profile was fully characterized. As expected, almost no data are available regarding the excipients as substrates of enzymes or transporters, and very limited data regarding their PK profile. The wide therapeutic margin may partially explain the lack of DDI studies of excipients as victim drugs but also the fact that many of these entities are not readily absorbed. The available in vitro and clinical data are discussed below.

### In vitro substrates

Only capsaicin had published data available evaluating the excipient as a substrate of metabolic enzymes. There were no studies identified evaluating any excipient of interest as a transporter substrate. Capsaicin is a substrate of multiple uridine 5′‐diphospho‐glucuronosyltransferases (UGTs) and does not appear to have been tested as a substrate of cytochrome P450 (CYP) enzymes. The published intrinsic clearance (CL_int_) values ranged from 0.79 to 19.6 µl/min/mg in UGT isoforms and was much higher in pooled human liver microsomes (142 µl/min/mg), indicating that capsaicin is subjected to significant hepatic glucuronidation. The highest affinity was observed for UGT1A7 and UGT2B15, with the K_m_ values of 13.1 and 13.9 µM, respectively.

### In vitro precipitants

The available in vitro data showed that the majority of excipients had been tested as precipitants as compared to substrates (21 compounds with metabolism and/or transport data as the precipitant vs. one compound with substrate data) with positive studies accounting for ~ 77% of both metabolism and transport studies.

For metabolism studies, CYP enzymes were the most commonly studied (65, majority being CYP3A) with the remaining studies being UGTs (21%) and other enzymes such as aldo‐keto reductases (15%). Most of the excipients with IC_50_ values were found to be weak inhibitors, reporting IC_50_ values greater than 10 µM. Only 17 of the 68 reported IC_50_ values (25%) were less than or equal to 10 µM and all were for two excipients—oleic acid and capsaicin. The most potent interactions for each were the inhibition of AKR1B10 by oleic acid (IC_50_ = 1.2 µM) and inhibition of CYP2C9 by capsaicin (IC_50_ = 2 µM). Other enzymes with potent interactions for these two excipients include CYP1A2, CYP2B6, and CYP2C9. On average, however, most reported inhibition constants were significantly higher (range = 1.2–950.1 µM, mean = 99.5 µM). It should be noted that this comparison is limited by differences in units, as only those reporting inhibition constants in µM were used (other units include mg/ml and volume per volume [%v/v]). Five excipients had induction data, reporting EC_50_ values ranging from 1.1 to 306.5 µM and changes in enzyme activity up to 2.5‐fold. Additionally, changes in mRNA of up to 4‐fold were observed for capsaicin. Among the excipients with EC_50_ values, cremophor EL was the most potent, showing an EC_50_ = 1.1 µM for CYP1A2. Cremophor EL was also found to inhibit in vitro CYP3A4, CYP2C9, and various UGTs (see Supplementary Data). Capsaicin was once again one of the most potent excipients evaluated for those reporting changes in activity, showing an approximate change in CYP3A activity of 2.5‐fold. Downregulation was also observed for some excipients with polysorbate 80 showing a change in CYP3A4 mRNA expression of 0.3‐ and 0.5‐fold in hepatocytes and Fa2N4 cells, respectively.

When transporters were evaluated, most experiments studied inhibition of P‐gp (36%) followed by MRP2 (14%), BCRP (4%), and OATP1B1 (6%). Only three studies evaluated induction potential of three compounds on MRP2 and none of them induced MRP2 mRNA or protein expression. All studies identified were completed with 19 excipients, with the majority of the studies for cremophor EL (*N* = 46), polysorbate 80 (*N* = 39), Solutol HS15 (*N* = 19), and d‐α‐Tocopherol polyethylene glycol 1000 succinate (*N* = 18). Among all the reported IC_50_ values, cremophor EL showed the highest potency for the interaction with OATP1A2 (IC_50_ = 0.00034% w/v). A majority of studies reported changes in uptake or efflux ratios (as appropriate) with the reported change ranging from 1.18‐ to 8.40‐fold. As was observed with reported IC_50_ values, the interaction of P‐gp with cremophor EL resulted in the greatest change in accumulation (substrate = daunorubicin, 8.4‐fold).^5^

### Clinical data

Only five excipients (5/37, 14%; diethylene glycol monoethyl ether EP/NF, cremophor EL, hydroxypropyl beta cyclodextrin, polysorbate 80, and sulfobutylether‐beta‐cyclodextrin) had published clinical PK data. Of the 35 studies available for these excipients, almost all were conducted in patients (83%) as opposed to healthy volunteers with the subjects being treated for renal impairment (varying stages, 53%) and various cancers (47%).

At this time, only clinical interaction data with the excipients of interest tested as inhibitors was identified through available literature (*N* = 22 studies). Half of the studies showed inhibition by the excipient, whereas the remaining studies either showed no effect (23%) or the interactions were attributed to other or complex mechanisms (changes to absorption, etc.; 27%). Although the site of interaction was not stated for many studies (8/21; 38%), P‐gp was the most frequently attributed target. Among the seven excipients (and one combination) that had clinical interaction data available, only three (3/7, 42%) had more than one study published. Furthermore, only one excipient (cremophor EL) had three or more EDI evaluations available in the literature. Most reported interactions were minor (median AUCR = 1.30), with 6 of 10 reaching clinical relevance as per the FDA criteria (AUCR ≥ 1.25). Co‐administration of cremophor EL yielded the highest change in substrate exposure, a 5‐fold increase in saquinavir AUC following oral dosing, most probably through inhibition of CYP3A and possible P‐gp.^6^ All other interactions resulted in an increase in AUC less than 2‐fold (1.28‐ to 1.59‐fold). Also included in the identified precipitant studies were seven interactions attributable to other mechanisms, such as changes in absorption.

### Case application of the knowledgebase

#### Model development

Based on the data evaluated, a fit‐for‐purpose model of cremophor EL was developed to illustrate the utility of the excipient database and to assist in better identifying the most relevant parameters to include in the knowledgebase for accurate modeling of these types of compounds. The aim of this modeling exercise was two‐fold: (i) to accurately model the PK of cremophor EL and (ii) to predict potential EDIs based on the information available in the knowledgebase. To maximize the utility of the limited clinical data available, development and verification datasets were established to ensure the accuracy of the model. A full description of model development and validation is presented in the Supplementary Methods.

Briefly, previously published clinical data on cremophor EL disposition were used for model development and validation.^7^, ^8^, ^9^, ^10^ When literature data were not available, such as for logP and pKa, values were estimated from the structure and/or physiochemical information identified in the knowledgebase. Model input parameters, with note of data identified from the knowledgebase, are presented in Table S1. All model development and testing were completed in Simcyp version 19 release 1. The primary assumption made based on the available data was that studies conducted in a patient population are representative of the population as a whole, as PK data in healthy volunteers were not available.

**FIGURE 1:**
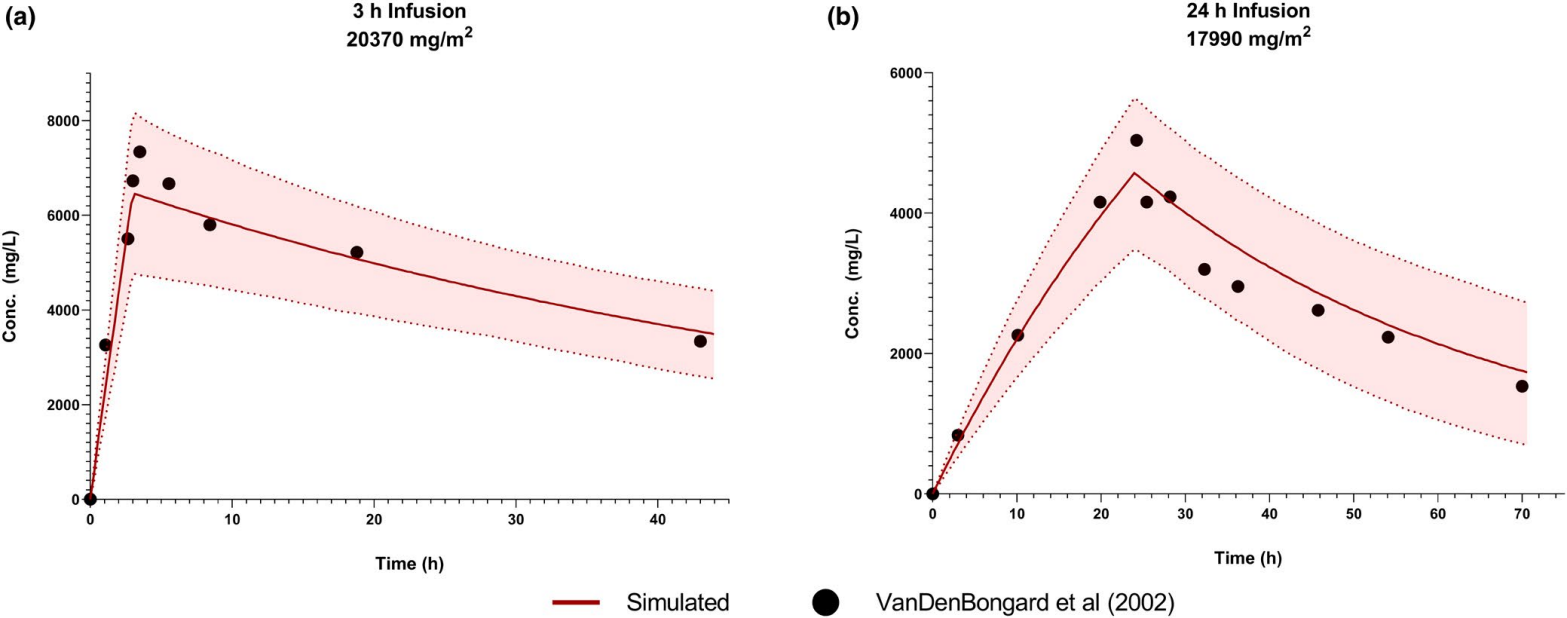
Simulated concentration versus time graphs with 95% confidence intervals for cremophor EL following 3‐h (a) and 24‐h (b) infusions. Literature data (dot) and simulated data (red line, mean value with 95% confidence interval)

A visual check was first completed to ensure the shape of the concentration‐time curve for the clinical and simulated data followed the same shape and acceptance criteria was preset to require model parameters to fall within ±30% of clinical values. For the development dataset the predicted AUC and C_max_ fell within 105% and 89%, respectively, of the literature data (Table 2). Additionally, all data points from the published study fell within the 95% confidence interval for the predicted concentration‐time data (Figure 1a).

**TABLE 2:**
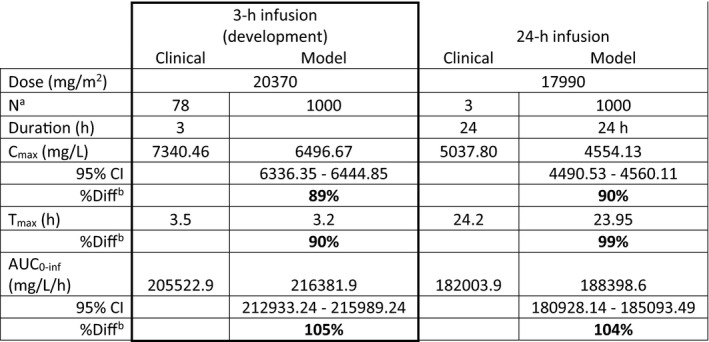
Cremophor EL PK parameters for model development and validation datasets

Note

Boxed data is the results of model development.

^a^ Number of subjects for clinical trial or simulation, respectively. Simulations used 100 subjects per trial for 10 trials.

^b^ %Diff = (predicted/observed)*100. Acceptance criteria is ±30% [70–130%]. Those parameters meeting the criteria are in bold text.

#### Model confirmation

The cremophor EL model was confirmed with literature data from a 24 h (*N* = 3 for the clinical data) infusion. The predicted C_max_ and AUC fell within 90% and 104%, respectively, and the shape of the curve was similar to the literature clinical data (Table 2, Figure 1b). Despite the limitations in available data, this exercise illustrated that the information currently available in the knowledgebase is adequate to build a minimal PBPK model for cremophor EL for infusions 24‐h or shorter.

#### Interaction modeling

Following validation of the cremophor EL model, the 3‐h infusion (20,370 mg/m^2^) was subsequently used to determine if sufficient data is present in the excipient knowledgebase to reasonably predict the risk of EDIs for common targets. Interactions of cremophor EL with CYP3A and P‐gp were modeled using published IC_50_ values for each (600 µM and 11.92 µM using testosterone and digoxin as the substrate, respectively).^11^, ^12^, ^13^ Midazolam and digoxin were used to test for the risk of CYP3A and P‐gp interactions, respectively, and the Simcyp compound files were used for each substrate. With simultaneous administration of intravenous cremophor EL and midazolam, an AUCR (inhibited / midazolam alone) of 3.8 was observed with no change in C_max_ (52.14 ng/ml vs. 51.90 ng/ml; Table S2). With digoxin as the substrate, evaluating interaction risk for P‐gp, AUC was increased 1.20‐fold compared to control. Although this does not reach the FDA criteria, a change in exposure of this magnitude might be clinically relevant due to the narrow therapeutic window of digoxin.

Whereas studies with these substrates and cremophor EL are not available in literature, similar interactions have been reported following oral administration of cremophor EL with fexofenadine (a known P‐gp substrate, AUCR = 1.30) and saquinavir (CYP3A and P‐gp; AUCR = 1.37–5.01).^6^, ^14^ The cremophor EL dose in these studies was lower than the dose used in the initial interaction modeling, therefore secondary simulations were run using 3‐h infusions of 100 mg and 5000 mg, lowest and highest oral doses reported, to determine if there is potential for an interaction at clinically relevant doses. For digoxin, there was little difference between doses with AUCRs of 1.14 and 1.20 for 100 mg and 5000 mg cremophor EL, respectively, whereas no difference in C_max_ was predicted. Interestingly, a dose‐dependent effect was observed in the change in midazolam exposure. At the lowest dose, there was no observed interaction (AUCR = 1.08) whereas the AUCR increased to 2.48 at the high dose. Furthermore, timing of administration does not appear affect the interaction, as delaying midazolam administration to the end of the 100 mg cremophor EL infusion resulted in a minimal change to the AUCR (1.15 vs. 1.08 for delayed and simultaneous administration, respectively; Table S2).

## DISCUSSION

Introduced here is a comprehensive repository of relevant information to enable quantitative predictions of drug interactions with commonly used excipients. Included data were identified through comprehensive queries of published data available through repositories, such as PubMed. Using the information from the knowledgebase, a fit‐for purpose minimal PBPK model for cremophor EL was developed illustrating the utility of the database and highlighting the potential risk of EDIs.

There are some limitations to this work. The knowledgebase relies on publicly available data to acquire parameter values of interest. Although it is likely that these excipients have been tested more extensively than the literature shows, the available information on this class of compounds is limited. This is highlighted, for example, in the limited number of excipients that had clinical PK data available and the similarly small number of compounds reporting in vitro disposition data. Research in this area appears to be in progress by various groups, with the lack of published data highlighting an interesting area of potential future work to better understand the disposition of these excipients, including the metabolic fate and interaction potentials in both healthy volunteers and patients.

This work highlights that there are significant gaps in the understanding of excipients themselves in addition to their role in interactions. Based on the areas of limited data acquired for the knowledgebase, the largest gaps in understanding at this time have been identified as physiochemical data (i.e., logP and protein binding), information on the compound as a substrate (both clinical and in vitro), and PK data in healthy volunteers. Physiochemical information is critical for modeling as it describes the compound and informs how it will interact with physiological processes. Despite the widespread use of these excipients in current formulations work, it appears that an understanding of the disposition of these compounds is essentially nonexistent in literature. For the limited excipients with relative more in vitro data, there are large variabilities in the reported values, possibly due to variables like assay systems, incubation conditions in performing the in vitro experiments among different laboratories. To help remedy this gap in knowledge and reduce the variability, work has been initiated by the Bill and Melinda Gates Foundation to conduct in vitro studies that will provide information on the potential of common excipients to alter metabolism and transport pathways.

Using the available data curated in this knowledgebase, a minimal PBPK model for cremophor EL was developed. Although the available physiochemical data for the compound were minimal, sufficient data was present to allow for estimation of key parameters including CL_IV_ and blood‐to‐plasma partitioning. However, the ability to apply the model to other dosing regimens and levels is lacking as the small clinical sample sizes and gaps in disposition data do not allow for complete extrapolation of the PK. The scarcity of data for excipients also leads to difficulties in accurately predicting changes in exposure for formulated drugs, shown here with the interaction modeling completed for CYP3A and P‐gp. Assessing the predictive ability of the model on interactions present in literature would be the ideal application, yet the lack of existing validated compound files for the substrates tested clinically limited possible applications. Therefore, these simulations using marker substrates are presented as a proof of concept only, as clinical data to confirm these observations is lacking. One key limitation is that for both pathways, only a single value of in vitro potency was available in literature, which does not allow for the ability to account for variability in K_i_ values and confirmation of assumed potency. Furthermore, increased reporting of excipient concentrations in clinical interaction studies, as well as improved prediction of intestinal lumen concentration‐time profiles of these excipients, would serve to better inform model design and allow for model validation and eventually for prospective prediction of EDIs. Meanwhile, further research into the in vitro potency (both for inhibition and induction) will ensure that the magnitude of effect is accurately portrayed.

The knowledgebase presented here serves as the first work to create a PK knowledgebase on excipients that will support quantitative predictions, through computational modeling, of the risk of EDIs and a more mechanistic understanding of the role of excipients in affecting drug disposition.

## CONFLICT OF INTEREST

The authors declared no competing interests for this work.

## AUTHOR CONTRIBUTIONS

S.J.M., J.Y., and I.R.‐M. wrote the manuscript. S.J.M., J.Y., and I.R.‐M. designed the research. S.J.M., J.Y., Y.W., C.W., and I.R.‐M. performed the research. S.J.M., J.Y., Y.W., C.W., and I.R.‐M. analyzed the data.

## Supporting information

Supplementary Material

Supplementary Material

Supplementary Material
