# Supplementary material for: Excipient knowledgebase: Development of a comprehensive tool for understanding the disposition and interaction potential of common excipients
Source: CPT Pharmacometrics Syst Pharmacol. 2021 Aug 1;10(8):953–61. doi: 10.1002/psp4.12668 (PMC8376134; doi:10.1002/psp4.12668)
Supplement: Supplementary file 1 — Supplementary Material [file PSP4-10-953-s002.docx]

**Excipient Data Collection SOP**

Sources: Drugs@FDA (published new drug application (NDA) reviews and drug labeling), Goodman and Gilman, chemical property databases/references (e.g. PubChem, ChemIDplus), material safety data sheets (MSDS), and other peer-reviewed papers identified through web queries (PubMed, Medline, etc.).

1. Query Format
   - Searches were performed for the following:
     - [excipient] clinical pharmacokinetics
     - [excipient] in vitro metabolism transport
     - [excipient] drug-drug interaction
2. Compound Parameters
   - Compound Identifier
     - CAS number will be included for each entry when available.
     - The substance name will be listed (“NAME”) as well as any additional common names (“SYNONYMS”) as appropriate.
     - Substance will be classified as a single entity or mixture as appropriate.
   - Physiochemical and Drug Properties
     - Parameters may be taken from any source listed previously, references should be clearly indicated.
     - A single unit should be used for each parameter when possible, indicate clearly when a different unit is used.
3. Clinical Pharmacokinetics
   - All available clinical PK data should be included.
     - Priority is given to tabulated data, when not available graphs can be digitized or parameters estimated from the figures if noted in the entry.
   - When provided, the population of the study conducted (patients, healthy volunteers, male/female) and the number of subjects should be recorded.
   - When additional information is provided, such as urinary recovery, data should be included in the notes column.
4. *In Vitro* Metabolism and Transport
   - Data for all metabolic enzymes/transporters described in the FDA, EMA, and PMDA DDI guidance documents should be evaluated.
     - Those studies citing interactions through unspecified pathways (i.e. “CYP enzymes” or “uptake transporters”) should also be included.
   - All study data should be input as a new row.
     - Studies with multiple results reported should be entered separately, although multiple parameters from a single study (i.e. K_m_ and V_max_) can be input as a single entry
   - For negative data, describe as “not metabolized/transported” or “not inhibited/induced” as appropriate

- For all inhibition data, specify the inhibition type (competitive, non-competitive, mixed) if listed, otherwise describe as “not specified”.

1. Clinical Interactions
   - Prioritize studies completed with known clinical marker substrates/inhibitors/inducers.
     - When studies with marker compounds are not available, include any available data being sure to indicate the enzyme/transporter implicated in the interaction by the study authors.
   - When provided, the study design, population of the study conducted (patients, healthy volunteers, male/female), and the number of subjects should be recorded
   - Negative data (no reported interaction) should also be included.

**Supplemental Methods – Cremophor EL model**

Clinical data on cremophor EL disposition published by van den Bongard *et. al.* (2002) was used for model development (3 h IV infusion) and confirmation (24 h IV infusion). Graphs were digitized using WebPlotDigitizer to determine the concentration-time profile for cremophor EL. PK parameters for each dose group were calculated using the *NonCompart* package in R (script included below).^1–4^ Doses were converted from (mL/m^2^) to (mg) and plasma concentration data was converted from (mL/L) to (mg/L) for evaluation in alignment with dosing options available in Simcyp, using a density for cremophor EL of 1.4 mg/mL and a standard body surface area (BSA) of 1.7 m^2^.^5^ All model development and testing was completed in Simcyp version 19 release 1.

Due to a lack of published physiochemical data, some parameters required estimation for model development. The IV clearance (CL_IV_) was determined from the NCA and confirmed with the parameter estimation tool in Simcyp using two published studies with data available for a 3-h infusion.^4,6^ Chemicalize was used to determine the logP and pKa from the structure (developed by ChemAxon, www.chemicalize.com).^7^ Fraction unbound was estimated in Simcyp and a standard blood-to-plasma ratio for acidic compounds was used in all predictions. Default simulator values were used for the single adjusting compartment in the minimal PBPK model (k_in_ and k_out_) as well as for volume (V_sac_).

As part of the model development, the key assumption made based on the available data was that studies conducted in a patient population are representative of the population as a whole, as PK data in healthy volunteers was not available. It was also assumed that a minimal PBPK model is appropriate for the available data and sufficient for the current needs, and that multiple IV infusions over the same time period result in the same profile as a single infusion. That is, three 1-h infusions result in the same exposure profile as one 3-h infusion. This is required due to dosing limitations within the Simcyp software as excipients are dosed in significantly larger amounts than most drugs.

To ensure an accurate model, a visual check was first completed to ensure the shape of the concentration-time curve for the clinical and simulated data followed the same shape and acceptance criteria was preset to require model parameters to fall within ± 30% of clinical values. Parameters for the cremophor EL compound file are included in **Table S1**. For the development dataset (3-h infusion of 14.55 mL/m^2^), predicted AUC and C_max_ fell within 105% and 89%, respectively, of the observed clinical data. Additionally, all clinical data points fall within the 95% confidence interval for the predicted concentration-time data and the two datasets show almost identical curve shape.

*Model Validation and Interaction Modeling*

The cremophor EL model was confirmed using the available 24 h (N = 3 for the clinical data) infusion data. While a 96-h infusion was also available, it was noted through the NCA that the clearance of the was significantly larger than for the lower dose groups, (CL_IV_ = 0.08 and 0.82 for the 24-h and 96-h infusions, respectively). Additionally, the volume of distribution was significantly higher for the 96-h infusion, and parameter estimation was used to identify an appropriate volume relative to the initial model value. Therefore it was decided that there was insufficient data to explain the difference and only the 24-h data would be used for confirmation.

The simulated C_max_ and AUC for the 24-h infusion fell within 90% 104% of the literature dataset, respectively. Additionally, the shape of the simulated curve was similar to that of the reported literature study. From this, it was determined that the data currently available is adequate to build a minimal PBPK model for cremophor EL for infusions 24-h or shorter.

Following validation of the cremophor EL model, the 3-h infusion (20,370 mg/m^2^) was used to determine if sufficient data is present in the excipient knowledgebase to reasonably predict the risk of EDIs for common targets. Interactions of cremophor EL with CYP3A and P-gp were modeled using published IC_50_ values for each (600 µM and 11.92 µM using testosterone and digoxin as the substrate, respectively) which were converted to K_i_ values using the Cheng-Prusoff equation, assuming that the concentration of substrate used is approximately equal to the K_m_ in the test system.^8–10^ Midazolam and digoxin were used to test for the risk of CYP3A and P-gp interactions, respectively, and the unaltered Simcyp compound files were used for each substrate. With simultaneous administration of intravenous cremophor EL and midazolam, an AUCR (inhibited / midazolam alone) of 3.8 was observed with no change in C_max_ (52.14 ng/mL versus 51.90 ng/mL; **Table S2**). With digoxin as the substrate, evaluating interaction risk for P-gp, AUC was increased 1.20-fold compared to control. While this does not reach the FDA criteria, a change in exposure of this magnitude might be clinically relevant due to the narrow therapeutic window of digoxin.

While studies with these substrates and cremophor EL are not available in literature, similar interactions have been reported following oral administration of cremophor EL with fexofenadine (a known P-gp substrate) and saquinavir (CYP3A and P-gp). In these studies, the reported change in AUC for fexofenadine was 1.30-fold while saquinavir showed changes of 1.37- to 5.01-fold as the cremophor EL dose increased.^3,11^ The cremophor dose in these studies was lower than the dose used in the initial interaction modeling, therefore secondary simulations were run using 3-h infusions of 100 mg and 5,000 mg, lowest and highest oral doses reported, to determine if there is potential for an interaction at clinically relevant doses. As was observed with the initial simulations, there was no change in C_max_ for either substrate (C_max_ ratio = 1.0 for both). For digoxin, there was little difference between doses with AUCRs of 1.14 and 1.20 for 100 mg and 5000 mg cremophor EL, respectively. Interestingly, a dose-dependent effect was observed in the change in midazolam exposure. At the lowest dose, there was no observed interaction (AUCR = 1.08) while the AUCR increased to 2.48 at the high dose. Furthermore, timing of administration does not appear affect the interaction, as delaying midazolam administration to the end of the 100 mg cremophor EL infusion resulted in a minimal change to the AUCR (1.15 versus 1.08 for delayed and simultaneous administration, respectively; Table 2).

Table S1. Compound Parameters for Cremophor EL

|  | **Value** | **Source** |
| --- | --- | --- |
| **Phys Chem and Blood Binding** | | |
| Mol Weight (g/mol) | 136.200 | Excipient database |
| log P | -1.839 | Predicted from structure |
| Compound Type | Monoprotic Acid | Excipient database |
| pKa | 13.610 | Predicted from structure |
| B/P | 0.630 | Predicted |
| fu | 0.953 | Simcyp estimate |
| Hematocrit | 45 | Simcyp default |
| **Distribution** | | |
| Model | Minimal PBPK Model |  |
| Vss (L/kg) | 0.070 | NCA |
| Vss %CV | 22.4% |  |
| k_in_ (-h) | 1.85E-06 | Simcyp default |
| k_out_ (-h) | 0.012 |  |
| **Elimination** | | |
| Clearance Type | In Vivo Clearance |  |
| CL_IV_ (L/h) | 0.07 | NCA |
| CL_IV_ %CV | 33.7 |  |
| CL R (L/h) | 0.070 | Simcyp default |
| **Transporters - Interaction** | | |
| P-gp Ki (µM) | 5.960 | Excipient database |
| fuinc (Ki) | 1.000 | Simcyp default |
| **CYPs and/or UGTs Interaction** | | |
| CYP2C9 Ki (µM) | 15.000 | Excipient database |
| CYP3A4 Ki (µM) | 300.000 | Excipient database |
| fu mic | 1.000 | Simcyp default |

Table S2. Summary of changes in substrate exposure following coadministration of cremophor EL to illustrate potential for excipient-drug interactions for CYP3A and P-gp.

|  | Midazolam (3 mg, IV) | | | | Digoxin (0.5 mg, IV) | | |
| --- | --- | --- | --- | --- | --- | --- | --- |
| Cremophor EL Dose | 20,370 mg/m^2^ | 100 mg | 100 mg^a^ | 5,000 mg | 20,370 mg/m^2^ | 100 mg | 5,000 mg |
| C_max_ Ratio | 1.00 | 0.99 | 0.99 | 1.00 | 1.00 | 1.00 | 1.00 |
| AUC_0-inf_ Ratio | **3.79** | 1.08 | 1.15 | **2.48** | 1.20 | 1.14 | 1.20 |

^a^Midazolam administered at the conclusion of the cremophor EL infusion. All other simulations were run with simultaneous dosing of the substrate and excipient.

Geometric mean values from the simulations are reported. Values in bold are at or above the FDA interaction threshold of 1.25-fold change.

References

1. R Core Team R: A language and environment for statistical computing. R Foundation for Statistical Computing, Vienna, Austria. (2019).at <https://www.r-project.org/>

2. Bae, K.-S. NonCompart: Noncompartmental Analysis for Pharmacokinetic Data. R package version 0.4.5. (2019).at <https://cran.r-project.org/package=NonCompart>

3. Rohatgi, A. WebPlotDigitizer (v 4.2). (2019).at <https://automeris.io/WebPlotDigitizer>

4. Bongard, H. Van den, Mathôt, R., Tellingen, O. Van, Schellens, J. & Beijnen, J. A population analysis of the pharmacokinetics of Cremophor EL using nonlinear mixed-effect modelling. *Cancer Chemother. Pharmacol.* **50**, 16–24 (2002).

5. ChemSrc. at <https://www.chemsrc.com/en/cas/61791-12-6_1376155.html>

6. Rischin, D. *et al.* Cremophor pharmacokinetics in patients receiving 3-, 6-, and 24-hour infusions of paclitaxel. *J. Natl. Cancer Inst.* **88**, 1297–301 (1996).

7. ChemAxon Chemicalize. (2020).at <https://www.chemaxon.com>

8. Cheng, Y. & Prusoff, W. H. Relationship between the inhibition constant (K1) and the concentration of inhibitor which causes 50 per cent inhibition (I50) of an enzymatic reaction. *Biochem. Pharmacol.* **22**, 3099–108 (1973).

9. Gurjar, R. *et al.* Inhibitory Effects of Commonly Used Excipients on P-Glycoprotein in Vitro. *Mol. Pharm.* **15**, 4835–4842 (2018).

10. Christiansen, A., Backensfeld, T., Denner, K. & Weitschies, W. Effects of non-ionic surfactants on cytochrome P450-mediated metabolism in vitro. *Eur. J. Pharm. Biopharm.* **78**, 166–72 (2011).
