# Supplementary material for: Excipient knowledgebase: Development of a comprehensive tool for understanding the disposition and interaction potential of common excipients
Source: CPT Pharmacometrics Syst Pharmacol. 2021 Aug 1;10(8):953–61. doi: 10.1002/psp4.12668 (PMC8376134; doi:10.1002/psp4.12668)
Supplement: Supplementary file 2 — Supplementary Material [file PSP4-10-953-s001.docx]

**Table S3. Knowledgebase Column Headings**

| **Compound Parameters** | **Clinical PK** | ***In Vitro* Substrate** | ***In Vitro* Precipitant** | **Clinical Interaction** |
| --- | --- | --- | --- | --- |
| Mixture / Single Substance | Population | Overall Effect | Overall Effect | Type of Study (marker/co-med) |
| Type (acid/base/zwitterion) | Population Descriptor(s) | Enzyme | Inhibition Type | Enzymes/Transporter(s) Implicated |
| B-P ratio | Diagnosis | Transporter | Enzyme | Population |
| F | # of subjects in test group | System | Transporter | Population Descriptor(s) |
| f_a_ | Study Design | K_m_ | System | Diagnosis |
| f_u,p_ | Route of Admin. | V_max_ | Precipitant Concentration | # of subjects in test group |
| k_a_ | Dose | CL_int_ | Substrate | Study Design |
| log_P_ | Frequency | Uptake Ratio | Substrate Concentration | Substrate |
| MW | Duration | Efflux Ratio | Metabolite | Substrate Route of Admin. |
| P_eff_ | C_ma_x | Other Parameters | Pre-Incubation | Substrate Dose |
| pKa | T_max_ | Comments | K_i_ | Substrate Frequency |
|  | t_1/2_ |  | IC_50_ | Substrate Duration |
|  | AUC_(0-inf)_ |  | EC_50_ | Precipitant Route of Admin. |
|  | CL_total_ |  | Percent Inhibition | Precipitant Dose |
|  | CL_renal_ |  | Fold Increase (Induction/Activation) | Precipitant Frequency |
|  | V_d_ |  | Other Parameters | Precipitant Duration |
|  | Study Notes |  | Comments | Precipitant Formulation |
|  |  |  |  | AUCR |
|  |  |  |  | CL Ratio |
|  |  |  |  | C_max_ Ratio |
|  |  |  |  | T_max_ Ratio |
|  |  |  |  | T_1/2_ Ratio |
|  |  |  |  | Other Parameters |
|  |  |  |  | Study Notes |
